# Insight into selective breeding for robustness based on field survival records: New genetic evaluation of survival traits in pacific white shrimp (*Penaeus vannamei*) breeding line

**DOI:** 10.3389/fgene.2022.1018568

**Published:** 2022-10-13

**Authors:** Shengjie Ren, Peter B. Mather, Binguo Tang, David A. Hurwood

**Affiliations:** ^1^ Faculty of Science, Queensland University of Technology, Brisbane, QLD, Australia; ^2^ Beijing Shuishiji Biotechnology Co., Ltd., Beijing, China

**Keywords:** *Penaeus vannamei*, survival traits, genetic parameters, heritability, genetic evaluation, selective breeding

## Abstract

Survival can be considered a relatively ‘old’ trait in animal breeding, yet commonly neglected in aquaculture breeding because of the simple binary records and generally low heritability estimates. Developing routine genetic evaluation systems for survival traits however, will be important for breeding robust strains based on valuable field survival data. In the current study, linear multivariate animal model (LMA) was used for the genetic analysis of survival records from 2-year classes (BL2019 and BL2020) of pacific white shrimp (*Penaeus vannamei*) breeding lines with data collection of 52, 248 individuals from 481 fullsib families. During grow-out test period, 10 days intervals of survival data were considered as separate traits. Two survival definitions, binary survivability (S) and continuous survival in days (SL), were used for the genetic analysis of survival records to investigate; 1) whether adding more survival time information could improve estimation of genetic parameters; 2) the trajectory of survival heritability across time, and 3) patterns of genetic correlations of survival traits across time. Levels of heritability estimates for both S and SL were low (0.005–0.076), while heritability for survival day number was found to be similar with that of binary records at each observation time and were highly genetically correlated (*r*_*g*_ > 0.8). Heritability estimates of body weight (BW) for BL2019 and BL2020 were 0.486 and 0.373, respectively. Trajectories of survival heritability showed a gradual increase across the grow-out test period but slowed or reached a plateau during the later grow-out test period. Genetic correlations among survival traits in the grow-out tests were moderate to high, and the closer the times were between estimates, the higher were their genetic correlations. In contrast, genetic correlations between both survival traits and body weight were low but positive. Here we provide the first report on the trajectory of heritability estimates for survival traits across grow-out stage in aquaculture. Results will be useful for developing robust improved pacific white shrimp culture strains in selective breeding programs based on field survival data.

## Introduction

Aquaculture is playing an increasingly important role in world food security, development of economic sustainability, and to provide practical solutions for addressing ecosystem services issues (Bernatchez et al., 2017; Houston et al., 2020; Naylor et al., 2021). Sustainability of aquaculture production in global food systems however, has become vulnerable due to the rapid expansion of aquaculture industry, outbreak of diseases/pathogens, water environmental pollution, and in particular, from the impacts of climate changes (Troell et al., 2014; Reid et al., 2019). Consequently, there has seen an increasing demand for better management and breeding of robust culture lines (Friggens et al., 2017). Genetic improvement *via* selective breeding is widely acknowledged as an efficient tool to improve economically important traits including: feed conversion ratios, biomass production, and overall survival rates of domesticated aquatic animals (Gjedrem et al., 2012; Hung et al., 2013; Nguyen, 2016; Gjedrem and Rye, 2018). A wide range of projects have confirmed selective breeding to be an efficient strategy for enhancing overall survival rate and disease resistance performance in farmed aquatic species, and thereby contributing to the development of aquaculture in a sustainable way (Yáñez et al., 2014; Houston, 2017).

In general terms, two selection approaches have been commonly used to develop robustness in aquaculture genetic breeding programs. One approach is to improve specific disease resistance *via* controlled challenge tests in target breeding lines. For this approach, tested families are artificially infected with a specific pathogen in a controlled environment condition *via* intra-peritoneal injection, immersion or cohabitation. Following this, additive genetic variance among families for phenotypic resistance against the target pathogen can be identified and used in future breeding plans (Ødegård et al., 2011; Yáñez et al., 2014). Currently, disease resistance strains have been developed successfully *via* this approach for a number of aquatic species, including farmed salmonid species (Correa et al., 2015; Vallejo et al., 2017; Barría et al., 2019), Pacific oyster (Gutierrez et al., 2018), and European sea bass (Palaiokostas et al., 2018). A second approach is to use selection based on survival data records in the field and this can provide another important data source for selecting robustness by improving overall individual survival rate (Gjedrem and Rye, 2018) and for developing specific disease resistance strains (Barría et al., 2020; Fraslin et al., 2022). This approach allows direct collection of data under real commercial farm conditions, and thereby avoids potential for genotype-by-environment (G-by-E) problems as seen in controlled challenge experiments between challenge test environments and production conditions on farm. Survival data records however, are often neglected in aquaculture breeding programs because they are based on simple binary records (0 or 1) and generally show low heritability.

Genetic analyses of survival phenotypic data in aquaculture breeding programs are commonly treated as a binary trait with ‘alive vs. dead’ reported for individuals at a specific observation time point scored as 1 and 0, respectively. Under this scenario, individuals that died early or later during the grow-out period would be given the same score, which means that useful information about relative survival time and/or lifespan is lost and therefore has not been used in the genetic analysis (Ødegård et al., 2006). Moreover, binary record variables of survival are commonly non-normally distributed, an issue that may compromise estimations of genetic components using a linear mixed animal model. To address this problem, survival records scored as continuous traits of survival time have been implemented successfully for genetic evaluation of survival data in animal breeding programs (Ducrocq and Casella, 1996; Van Pelt et al., 2015; Heise et al., 2016). In this way, survival phenotype can be recorded as normally distributed continuous traits and information about individuals with different survival time/lifespans can be captured effectively in the analysis. To date, genetic analyses of survival data scored as continuous traits have only been reported on farmed aquatic species for controlled challenge tests (Suebsong et al., 2019; Joshi et al., 2021a) while survival data from commercial breeding lines has rarely been investigated.

Different genetic evaluation models are available for genetic analyses of survival traits in animal breeding (Forabosco et al., 2009). A proportional hazard model (PHM) is a common model used for genetic evaluation of survival traits, and can be applied with a free open access software package in *Survival Kit* (Ducrocq and Sölkner, 1994; Ducrocq et al., 2010). PHM can handle large survival data sets rapidly and easily, manage survival data sets with skewed distributions, and is considered to provide accurate estimates (Sewalem et al., 2010; Zavadilová et al., 2011). In genetic evaluations however, it is difficult to process genetic correlations with other continuous traits at the same time (Tarrés et al., 2006). Alternatively, linear models and threshold models have been widely used for genetic analyses of survival data in aquaculture genetics. Compared with linear models, threshold models applying a logit link function are feasible for dealing with non-normally distributed binary survival data. However, they generally require more computational time and cannot estimate genetic correlations with other continuous traits simultaneously. In practice, analyses using either threshold models or PHM take almost five to ten times more computational time than do applying linear models to address almost the same tasks (Boettcher et al., 1999). Moreover, estimations of true breeding values (EBVs) have shown very similar correlations between linear models and threshold models (Veerkamp et al., 2002). While different linear models including random regression models (RRMs) have been used successfully for national genetic evaluations of survival data in dairy cattle (Sasaki et al., 2015; Van Pelt et al., 2015, 2016; Heise et al., 2016, 2018), there are currently no standardized model choices for routine genetic evaluation of survival data in aquaculture breeding programs.

Another important consideration for genetic analysis of survival traits in aquaculture species is changes of time. Survival traits are dynamic quantitative traits, which can change spatially and temporally due to multiple interactions between animal and environmental constraints. Therefore, understanding the trajectory of time for survival traits can be very useful for making critical decisions in a breeding plan (Schaeffer, 2004). Studies on trajectory of time for important economic traits in aquaculture genetics are still rare (Vehviläinen et al., 2010), and the few reported cases have mostly focused on growth traits (Turra et al., 2012; He et al., 2017; Schlicht et al., 2018), while to date, there has been no reports on trajectory of survival traits across grow-out testing stage.

Pacific white shrimp (*Penaeus vannamei*) has become the most widely farmed prawn species across the world. Annual global production reached ∼4.4 million tons with a commercial value of 26.7 billion USD in 2020, which ranked as the most important traded food commodity across the aquaculture sector (Kumar and Engle, 2016; FAO, 2020). Sustainability of prawn farming has been affected however, by emergence of several diseases that show high mortality rates (Robinson et al., 2022). Developing robust culture strains of pacific shrimp *via* selective breeding will play a crucial role for improving the economic profitability and animal welfare of this major aquatic farmed species. The main objective here was therefore to develop routine genetic evaluation of survival records in a genetic improvement program for pacific shrimp in China. Specifically, we evaluated: 1) genetic parameters from two different survival definitions for the binary traits of survivability and the continuous trait of survival time; 2) the trajectory of heritability for survival traits across the grow-out test period; and 3) genetic correlation patterns for survival traits across grow-out stage and their correlation with growth. Results from the current program will provide insight into selection breeding of robust culture strains based on field survival records in aquaculture species.

## Materials and methods

### Study population

The study population constituted the breeding nucleus from a pacific white shrimp stock improvement program in Hainan Island (China), with the selection target being for local farm environments in China based on a family selection approach. Foundation populations were produced in 2015 sourced from 12 hatchery lines in China representing four genetic populations as evidenced from a population structure analysis (Ren et al., 2018). In 2015, 98 full families were produced (Ren et al., 2020a). Following this, for each breeding cycle, 209–250 families were produced over a 1-week period. Family pedigree management used physical visible implant elastomer (VIE) tags, while in parallel a parentage assignment panel (Ren et al., 2022) was developed in 2019 to meet the demands of more large family numbers in the selective breeding program. The mating system for this program used a nested mating design *via* one single male with two females. The ratio of dam/sire was maintained at ∼ 1.7 with the aim to generate better genetic tier for EBVs estimation. Grow-out management conditions of the breeding line have been reported in earlier studies (Ren et al., 2020b, Ren et al., 2020c).

### Data records

In the current study, genetic analysis data were sourced from two different year classes 2019 (BL 2019) and 2020 (BL 2020) in the breeding nucleus line. The BL2019 line, consisted of 243 full-sibs families generated from 157 sires and 243 dams over a 1-week period, while the BL2020 line consisted of 238 full-sibs families produced by 143 sires and 238 dams (Table 1). The mean number of shrimp per family for the grow-out test was 102.8 for BL2019 and 114.7 for BL2020, with a total number of 52, 248 individuals recorded for data collection (Table 1).

**TABLE 1:** Data structure of pacific white shrimp breeding nucleus lines.

**Year**	**No. Shrimp**	**Shrimp/family**	**No. Family**	**Sires**	**Dams**
2019	24, 980	102.8	243	157	243
2020	27, 304	114.7	238	143	238
Total/mean	52, 284	108.7	481	300	481

Dead individuals from the breeding lines were collected three times per day across the grow-out period and pedigree information of mortalities was recorded from visible implant elastomer (VIE) tags. At the end of the grow-out test stage, all remaining harvested individuals in the test system were scored for body weight, pedigree of family ID, and gender.

### Trait definition

Data collected on survival from each 10 days interval were considered as separate traits over the grow-out period. Survival phenotype was recorded using two definitions in the genetic analysis. First, survival phenotype at the end of each 10 day period was coded as a binary trait of survivability, with 0 for dead individuals and 1 for live individuals. Secondly, survival data for each 10 day period was considered to be a continuous trait of survival time indicating how many days individuals survived over the test period. For example, live individuals in each breeding line at surviving to 100 days were coded as 100d and dead individuals at 86 days or 56 days were coded as 86d or 56d, respectively. Therefore, survival data records from BL2019 breeding line across the 117 days grow-out period were coded as 12 survivability binary traits (S1, S2, …, S11, S12) and 12 continuous traits of survival (SL1, SL2, …, SL11, SL12) for each observed time window of 10 days. Similarly, the 98 days records of survival data from BL2020 considered 10 survival binary traits (S1, S2, …, S9, S10) and 10 continuous traits of survival days (SL1, SL2, …, SL9, SL10), respectively. At the conclusion of the grow-out tests, phenotype of body weight for each shrimp was collected as a continuous trait (BW), while data from the final population census of live shrimps were recorded as a binary survival trait (SUR).

### Statistical analysis

#### Survival probability estimates and daily mortality

Changes in survival probability of shrimp in breeding lines across the experimental grow-out period were analysed using the Kaplan-Meier estimator (Kaplan and Meier, 1958). Shrimp that survived at each observed time (days) were treated as censored. The “Survminer” function (Kassambara et al., 2017) in the **R** package (R Core Team, 2019) was used to estimate Kaplan-Meier survival curves by;

$$\hat{S\;}\left(t\right)=\frac{\hat{\Pi }}{t_{i}\leq t}\;\left(\;1-\frac{d_{i}}{n_{i}}\;\right)$$

where, *n*_*i*_ is the number of alive shrimp at risk at observed time *t*_*i*_, and *d*_*i*_ is the number of deaths at the observed time. In addition, daily mortality in the breeding lines was also plotted for the general husbandry management assessment purposes.

### Genetic analyses

#### Genetic parameters

WOMBAT software (Meyer, 2007) was used to fit the following linear multivariate animal model (LMA) for the genetic analysis:

y=Xβ+Zα+e (1)

where, ***y*** is a vector of phenotype for the traits defined here (S1-S12, SL1-SL12, SUR, and BW); ***β*** is the vector of fixed effects including sex, tanks and family batches; ***α*** is the vector of random additive genetic effects; ***e*** is the vector of random residual errors; and, ***X*** and ***Z*** are known incidence matrices relating observations to the fixed and random effects mentioned above. Both vector of ***α*** and ***e*** are assumed to be multivariate normal distribution with mean zero and variances as:

$$Var\;\left[\begin{array}{c} \alpha \\ e \end{array}\right]=\left[\begin{array}{cc} A\sigma_{\alpha }^{2} & 0\\ 0 & {I\sigma }_{\alpha }^{2} \end{array}\right],$$

here, ***σ***_***α***_^***2***^ and ***σ***_***e***_^*2*^ are the random additive variances and error variances, respectively. **A** is the numerator relationship matrix based on pedigree information, and **I** represents an identity matrix. Total variance (*σ*_*p*_^*2*^) was calculated as the sum of random additive genetic variance (*σ*_*α*_^*2*^) and random residual components (*σ*_*e*_^*2*^). Heritability (*h* ^*2*^) was calculated as the ratio of the random genetic variance to the total phenotype variance, $h^{2}=\sigma_{\alpha }^{2}/\sigma_{p}^{2}$ . Phenotypic correlation (*r_p_*) between two traits was calculated as: $\gamma_{p}=\frac{\sigma_{\alpha 1,2}+\sigma_{e1,2}}{\sqrt{\left(\;\sigma_{\alpha 1}^{2}+\sigma_{e1}^{2}\right)+\left(\;\sigma_{\alpha 2}^{2}+\sigma_{e2}^{2}\right)}}$ ; and genetic correlation (*r*_*g*_) between two traits was calculated as: $\gamma_{g}=\frac{\sigma_{\alpha 1,2}\;}{\sqrt{\sigma_{a1}^{2}\;\sigma_{a2}^{2}}}$ . The values of data matrix for *r*_*p*_ and *r*_*g*_ were displayed as heat maps in **R** package (R Core Team, 2019). From the output of LMA genetic analyses, *r*_*p*_ and *r*_*g*_ between phenotype traits were aligned using **R** package in the ‘Matrix’ package and after, function of ‘pheatmap’ in R was used to plot the graphic representations of the correlation matrix data.

### Correlations of binary traits and survival days

Bivariate analyses were performed, in which the two definition types of survival phenotype were modeled simultaneously. Equations used in the bivariate animal model can be defined as follows:

$$\left[\begin{array}{c} y_{1}\\ y_{2} \end{array}\right]=\left[\begin{array}{cc} X & 0\\ 0 & X \end{array}\right]\;\left[\begin{array}{c} \beta_{1}\\ \beta_{2} \end{array}\right]+\left[\begin{array}{cc} Z & 0\\ 0 & Z \end{array}\right]\;\left[\begin{array}{c} \alpha_{1}\\ \alpha_{2} \end{array}\right]+\left[\begin{array}{c} e_{1}\\ e_{2} \end{array}\right]$$ (2)

where, the symbols represent the same vectors as described in the multivariate analysis of Model 1; the subscripts 1 and 2 are two different records for survival data of binary traits (S1-S12) and survival days (SL1-SL12) at each observed time window, respectively.

### Common environmental effect

Two linear univariate animal models were developed for the significance test of the full-sib family effect (*c* ^*2*^) in the genetic analyses. Equations of univariate animal models can be written as follows:

y=Xβ+Zα+Wf+e (3)

y=Xβ+Zα+e (4)

where, ***f*** is the vector of random full-sib family effects and **W** is the corresponding design matrices; other symbols represent the same vectors as described in Model 1. Compared with Model 3, there was no random full-sib family effect in Model 4. Likely statistical significance for the random full-sib family effects were analysed using a likelihood ratio approach. After running the two above univariate Models, significance for the random full-sib family effects were compared to the final log-likelihood (Maximum log L) using a Chi-square (*χ* ^*2*^) test.

## Results

### Patterns of survival

**FIGURE 1:**
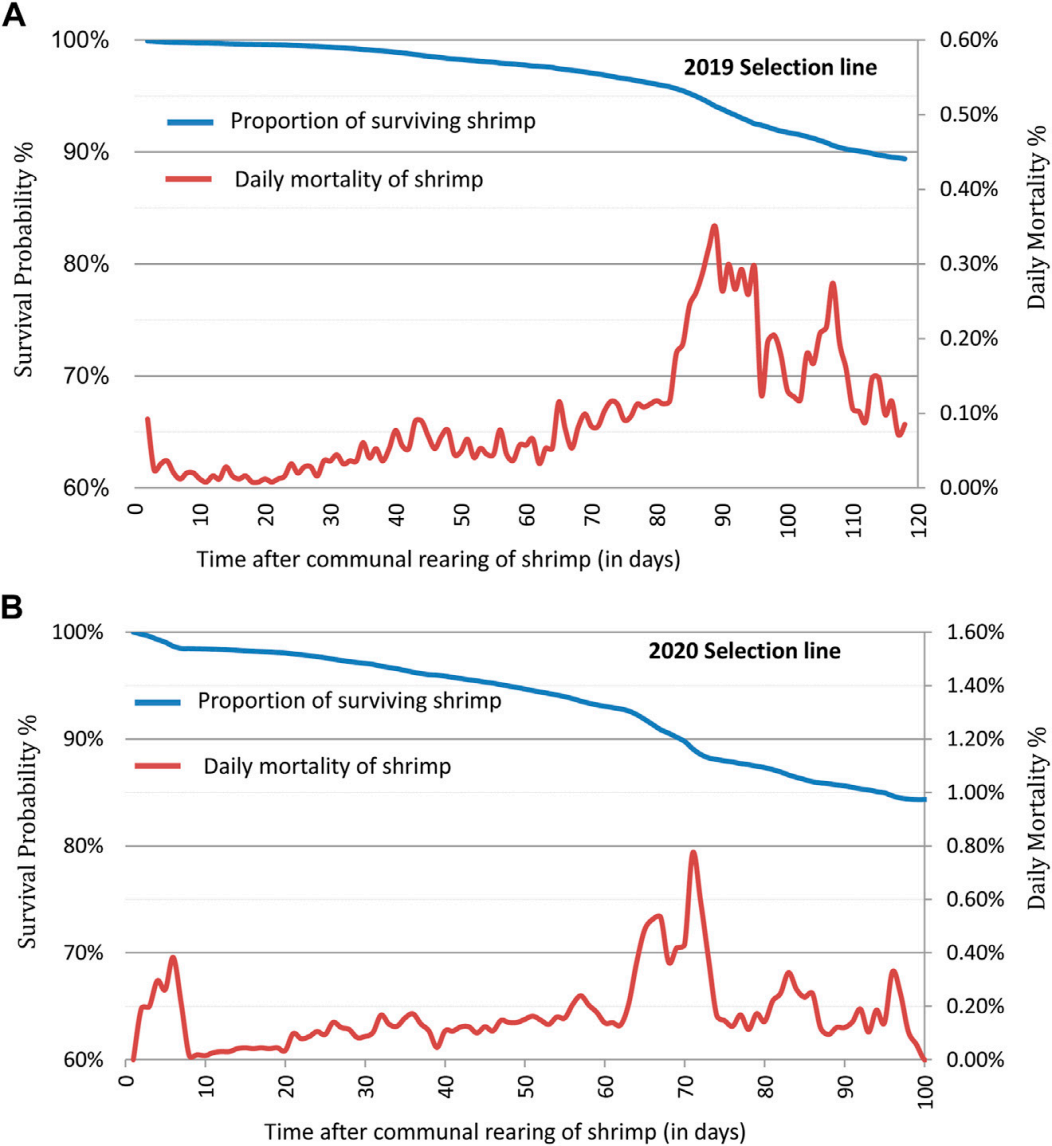
Kaplan-Meier survival curves and daily mortality changes for the shrimp breeding lines: **(A)** 2019 (BL 2019) of 243 fullsib families and **(B)** 2020 (BL 2020) of 238 fullsib families.

Kaplan–Meier survival curves showed changes in survival probability across the grow-out test periods for each year breeding line over the 2 year test period (Figure 1). At the end of the grow-out test period, survival probability for BL2019 was 89.4%, while for BL2020 it was 84.4%. These survival estimators however, presented some bias to the final population census (80.4% and 64.2%) at the end of grow-out test. Differences between the bias estimates indicate a proportion of shrimp mortality events apparently were not detected over the test periods. There were also significant differences (*p* < 0.01) in mortality among families for the two breeding lines investigated here. Means of daily mortality records for BL2019 and BL2020 were 0.1% and 0.17%, respectively (Figure 1). Overall, daily mortalities fluctuated at 0.1%–0.25% for breeding lines across the 2 years. Small peaks in mortality were first observed at the beginning of each test in the first week, which in part, can be explained as due to handling stress during VIE tagging. Most daily mortality peaks were evident at later stages of the grow-out test periods, where the highest daily mortality at 0.38% peaked at ∼ 90 days for BL 2019, while the highest daily mortality for BL2020 peaked for 0.78% at 70 days. The time period of the highest daily mortality events coincided with the coldest winter season period at the hatchery location in Hainan.

### Descriptive statistics of genetic analyses

For both year breeding lines, all families had records for survival data. Basic descriptive statistics for each trait are presented in Table 2. The coefficient of variation (CV) for survival traits gradually increased for survival data record (SL) in both year lines. CV for survival days ranged from 3.32% to 12.49% for BL 2019, while similar patterns for CV of SL were also observed for the BL 2020 line. Means of survival trait (S) at the end of the grow-out test period were higher than that of SUR, indicating some mortality events had not been observed.

**TABLE 2:** Descriptive statistics of phenotype traits for the breeding lines of BL2019 and BL 2020.

**Trait**	***BL2019***				***BL2020***			
	***N***	**Min**	**Max**	**Mean**	***N***	**Min**	**Max**	**Mean**
S1	24,980	0.000	1.000	0.998	27,304	0.000	1.000	0.984
S2	24,980	0.000	1.000	0.996	27,304	0.000	1.000	0.981
S3	24,980	0.000	1.000	0.994	27,304	0.000	1.000	0.972
S4	24,980	0.000	1.000	0.990	27,304	0.000	1.000	0.959
S5	24,980	0.000	1.000	0.984	27,304	0.000	1.000	0.947
S6	24,980	0.000	1.000	0.979	27,304	0.000	1.000	0.932
S7	24,980	0.000	1.000	0.973	27,304	0.000	1.000	0.899
S8	24,980	0.000	1.000	0.963	27,304	0.000	1.000	0.876
S9	24,980	0.000	1.000	0.942	27,304	0.000	1.000	0.865
S10	24,980	0.000	1.000	0.922	27,304	0.000	1.000	0.859
S11	24,980	0.000	1.000	0.910	*NA*	*NA*	*NA*	*NA*
S12	24,980	0.000	1.000	0.903	*NA*	*NA*	*NA*	*NA*
SL1	24,980	1.000	10.000	9.986	27,304	1.000	10.000	9.904
SL2	24,980	1.000	20.000	19.957	27,304	1.000	20.000	19.730
SL3	24,980	1.000	30.000	29.909	27,304	1.000	30.000	29.493
SL4	24,980	1.000	40.000	39.833	27,304	1.000	40.000	39.134
SL5	24,980	1.000	50.000	49.704	27,304	1.000	50.000	48.657
SL6	24,980	1.000	60.000	59.521	27,304	1.000	60.000	58.045
SL7	24,980	1.000	70.000	69.288	27,304	1.000	70.000	67.215
SL8	24,980	1.000	80.000	78.978	27,304	1.000	80.000	76.058
SL9	24,980	1.000	90.000	88.535	27,304	1.000	90.000	84.753
SL10	24,980	1.000	100.000	97.862	27,304	1.000	98.000	93.352
SL11	24,980	1.000	110.000	107.024	*NA*	*NA*	*NA*	*NA*
SL12	24,980	1.000	118.000	116.089	*NA*	*NA*	*NA*	*NA*
SUR	24,980	0.000	1.000	0.755	27,304	0.000	1.000	0.585
BW	20,078	2.300	57.000	21.394	17,529	1.900	41.900	17.946

*N*, the number of shrimp; S1-S12, survivability of binary traits for each observed time window of 10 days period; SL1-SL12, continuous traits of survival days for each observed time window of 10 days period; SUR, binary trait of survival for the final population census; BW, body weight of shrimp.

### Genetic parameters

**FIGURE 2:**
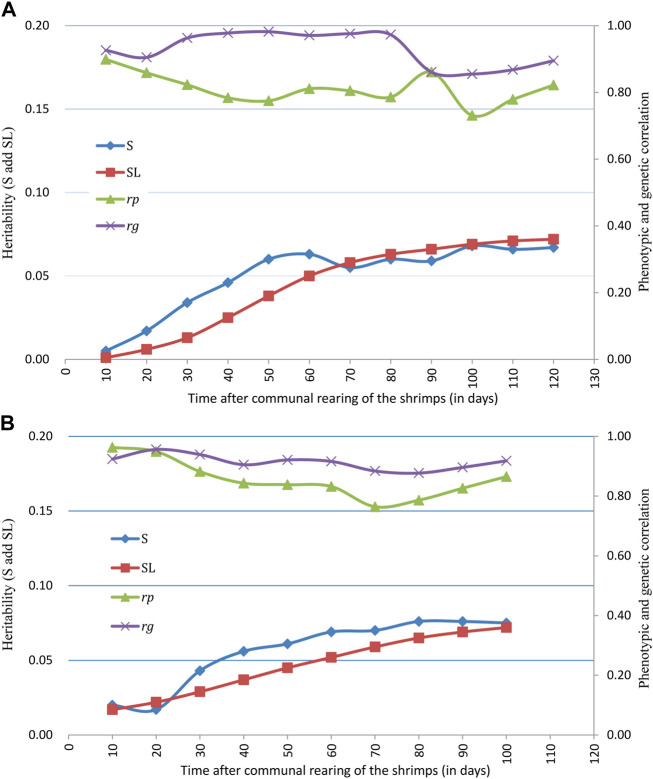
Trajectories of heritability for survival traits (S and SL), phenotypic correlation (*r*_*p*_) and genetic correlation (*r*_*g*_) between S and SL across grow-out test period of **(A)** BL2019 line, **(B)** BL2020 line.

Estimates of variance components and heritability of survivability records for binary traits (S), survival days (SL), final population census of survival (SUR), and body weight (BW) are presented in Table 3. Levels of heritability estimates for S and SL were both low and ranged from 0.005 to 0.076. Heritability for survival days (SL) was found to be closer with that of binary records (S) at each time of observation. While slightly higher heritability estimates were found for S than for SL at early stages of the grow-out testing period in the BL2019 line, they were very closer in the middle to later test periods. The trajectories of S and SL during grow-out test period are illustrated in Figure 2. The slightly higher heritability estimates for survival in BL2020 than in BL2019 were expected since we recorded a higher daily mortality in BL 2020. It was interesting that, for both 2-year data groups, heritability of S showed a steady increase over time. While SL estimates increased during the first 60 days, levels of heritability for SL fluctuated at ∼0.06 and maintained a plateau after this initial period.

**TABLE 3:** Estimates of variance components and heritabilities (*h* ^*2*^ *± se*) for the genetic analyse of survival data and body weight.

Trait	*BL2019*				*BL2020*			
	*σ*_*α*_^*2*^	*σ*_*p*_^*2*^	*σ*_*e*_^*2*^	*h* ^*2*^ *± se*	*σ*_*α*_^*2*^	*σ*_*p*_^*2*^	*σ*_*e*_^*2*^	*h* ^*2*^ *± se*
S1	1.09 E^-5^	2.31 E^-3^	2.30 E^-3^	0.005 *±* 0.002	3.02 E^-4^	1.53 E^-2^	1.50 E^-2^	0.020 *±* 0.004
S2	6.64 E^-5^	3.83 E^-3^	3.76 E^-3^	0.017 *±* 0.003	5.88 E^-4^	1.86 E^-2^	1.80 E^-2^	0.017 *±* 0.003
S3	2.11 E^-4^	6.21 E^-3^	6.00 E^-3^	0.034 *±* 0.005	1.16 E^-3^	2.70 E^-2^	2.58 E^-2^	0.043 *±* 0.006
S4	4.59 E^-4^	9.99 E^-3^	9.53 E^-3^	0.046 *±* 0.005	2.20 E^-3^	3.96 E^-2^	3.74 E^-2^	0.056 *±* 0.007
S5	9.57 E^-4^	1.58 E^-2^	1.49 E^-2^	0.060 *±* 0.007	3.11 E^-3^	5.08 E^-2^	4.77 E^-2^	0.061 *±* 0.007
S6	1.27 E^-3^	2.03 E^-2^	1.90 E^-2^	0.063 *±* 0.008	4.38 E^-3^	6.32 E^-2^	5.89 E^-2^	0.069 *±* 0.008
S7	1.45 E^-3^	2.62 E^-2^	2.48 E^-2^	0.055 *±* 0.007	6.31 E^-3^	9.06 E^-2^	8.43 E^-2^	0.070 *±* 0.008
S8	2.08 E^-3^	3.49 E^-2^	3.29 E^-2^	0.060 *±* 0.007	8.21 E^-3^	1.09 E^-1^	1.00 E^-1^	0.076 *±* 0.009
S9	3.16 E^-3^	5.38 E^-2^	5.07 E^-2^	0.059 *±* 0.007	8.88 E^-3^	1.17 E^-1^	1.08 E^-1^	0.076 *±* 0.009
S10	4.85 E^-3^	7.11 E^-2^	6.62 E^-2^	0.068 *±* 0.008	9.15 E^-3^	1.21 E^-1^	1.12 E^-1^	0.075 *±* 0.009
S11	5.32 E^-3^	8.08 E^-2^	7.55 E^-2^	0.066 *±* 0.008	*NA*	*NA*	*NA*	*NA*
S12	5.86 E^-3^	8.70 E^-2^	8.12 E^-2^	0.067 *±* 0.008	*NA*	*NA*	*NA*	*NA*
SL1	1.14 E^-4^	1.10 E^-1^	1.10 E^-1^	0.001 *±* 0.002	1.09 E^-2^	6.34 E^-1^	6.23 E^-1^	0.017 *±* 0.003
SL2	3.79 E^-3^	6.56 E^-1^	6.52 E^-1^	0.006 *±* 0.002	9.25 E^-2^	4.17 E^0^	4.08 E^0^	0.022 *±* 0.004
SL3	2.58 E^-2^	1.96 E^0^	1.93 E^0^	0.013 *±* 0.003	3.29 E^-1^	1.16 E^1^	1.13 E^1^	0.029 *±* 0.005
SL4	1.11 E^-1^	4.46 E^0^	4.35 E^0^	0.025 *±* 0.004	9.16 E^-1^	2.46 E^1^	2.37 E^1^	0.037 *±* 0.005
SL5	3.41 E^-1^	8.93 E^0^	8.58 E^0^	0.038 *±* 0.005	2.06 E^0^	4.55 E^1^	4.35 E^1^	0.045 *±* 0.006
SL6	8.28 E^-1^	1.65 E^1^	1.56 E^1^	0.050 *±* 0.007	4.01 E^0^	7.63 E^1^	7.23 E^1^	0.052 *±* 0.007
SL7	1.61 E^0^	2.80 E^1^	2.63 E^1^	0.058 *±* 0.007	7.05 E^0^	1.19 E^2^	1.12 E^2^	0.059 *±* 0.007
SL8	2.80 E^0^	4.45 E^1^	4.17 E^1^	0.063 *±* 0.008	1.16 E^1^	1.79 E^2^	1.67 E^2^	0.065 *±* 0.008
SL9	4.50 E^0^	6.78 E^1^	6.33 E^1^	0.066 *±* 0.008	1.79 E^1^	2.59 E^2^	2.41 E^2^	0.069 *±* 0.008
SL10	6.98 E^0^	1.01 E^2^	9.37 E^1^	0.069 *±* 0.008	2.60 E^1^	3.61 E^2^	3.35 E^2^	0.072 *±* 0.008
SL11	1.05 E^1^	1.47 E^2^	1.36 E^2^	0.071 *±* 0.008	*NA*	*NA*	*NA*	*NA*
SL12	1.50 E^1^	2.08 E^2^	1.93 E^2^	0.072 *±* 0.008	*NA*	*NA*	*NA*	*NA*
SUR	1.45 E^-2^	1.84 E^-1^	1.70 E^-1^	0.079 *±* 0.008	3.86 E^-2^	4.89 E^-1^	4.50 E^-1^	0.079 *±* 0.003
BW	1.59 E^0^	3.34 E^1^	1.75 E^1^	0.486 *±* 0.036	6.69 E^0^	1.79 E^1^	1.12 E^1^	0.373 *±* 0.031

*Note: σ_α_^2^, σ_p_^2^, and σ_e_^2^* represent additive genetic variance, phenotype variance, and residual variance; S1-S12, SL1-SL12, SUR, and BW: see legend in Table 2; *NA*, not applicable.

Heritability estimates for final population census survival (SUR) were very similar for S and SL at the final testing stage, while for body weight (BW) in BL2019 and BL 2020, they were 0.486 *±* 0.036 and 0.373 *±* 0.031, respectively.

### Phenotype and genetic correlations

**FIGURE 3:**
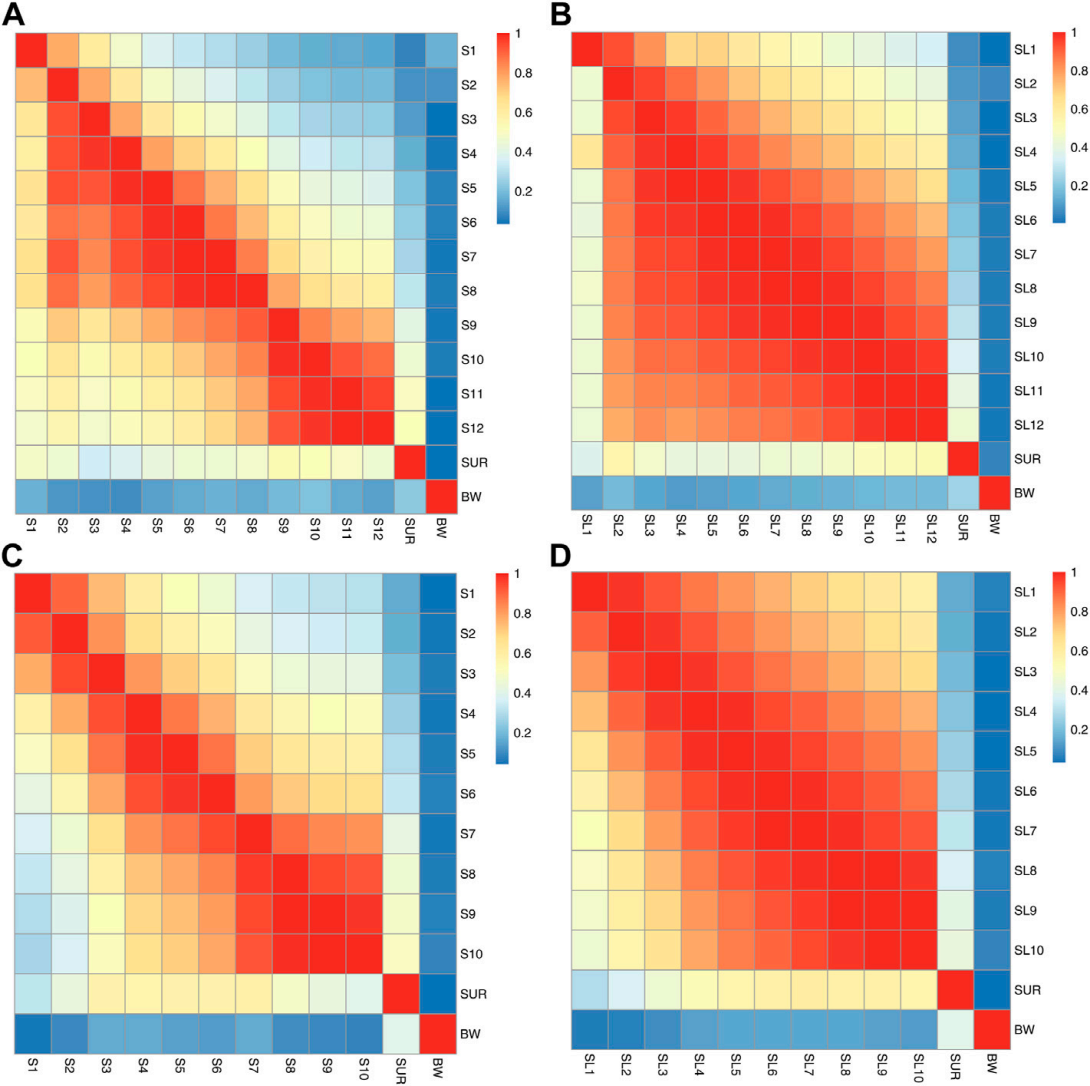
The trajectory of phenotype (above diagonal) and genetic correlations (below diagonal) between **(A)** S1-S12, SUR and BW for the breeding line of BL 2019, **(B)** SL1-SL12, SUR and BW of BL 2019, **(C)** S1-S10, SUR and BW for the breeding line of BL 2020, **(D)** SL1-SL10, SUR and BW for the breeding line of BL 2020.

Summaries of phenotype (*r*_*p*_) and genetic (*r*_*g*_) correlations with *SE* for S, SL, SUR, and BW are provided in Supplementary Tables S1–S4. In general *r*_*g*_ for S, estimates between different times were moderate to high and ranged from 0.475 (S1 *vs* S12) to 0.999 (S11 *vs* S12) in the BL2019 breeding line, and 0.277 (S1 *vs* S10) to 0.999 (S9 *vs* S10) for BL 2020, respectively. Estimates of *r*_*g*_ for SL between different times were much closer compared with that of S in both 2-year breeding lines. As expected, estimates of *r*_*g*_ between SUR and S, or SUR and SL were much lower, and in most cases were only moderate correlations likely due to the issues referred to unobserved mortality events when estimating SUR vs. S/SL. In contrast, estimates of *r*_*g*_ for BW and all survival related traits (S/SL/SUR) were low but all positive. We consider that this result implies that selection directed on survival related traits would not experience potentially negative tradeoff effects on growth traits. Patterns of phenotype correlations (*r*_*p*_) among S, SL, SUR, and BW were similar to those for *r*_*g*_ but were always slightly lower than for *r*_*g*_ (Figure 3).

Trajectory of correlations between S and SL estimates across the grow-out test period are illustrated in Figure 2. Genetic correlations between binary survival traits (S) and survival days (SL) were all high at the different observation times and ranged from 0.855 to 0.982 for BL2019 and 0.877 to 0.956 for BL 2020. The results reported above suggest that families with high survival rates are also likely to show a general trend for a longer mean life span over the test period. Trajectories of genetic correlations for S traits across time were consistent with that of estimation for SL, with estimates for *r*_*g*_ gradually decreasing across the experimental time period (Figure 3). Patterns of change on *r*_*g*_ estimates for S/SL across time were similar between breeding lines in both years.

### Full-sib family effects

The likelihood ratio test suggested that there were very limited full-sib family effects (*c* ^*2*^) in the genetic analysis. Among 26 comparison for BL2019, 24 tests were not significant for the likelihood ratio tests (Supplementary Table S5). In contrast, S1 and SUR were significant for *c* ^*2*^ (*χ* ^*2*^ *_1df_*). Data interpretation for genetic parameters for S1 and SUR however, showed limited potential for improving an animal model fit for full-sib family effects (Model 3) because the new results for S1 and SUR genetic parameters showed large associated SE estimates (S1, *h* ^*2*^ = 0.005 ± 0.009; SUR, *h* ^*2*^ = 0.053 ± 0.070). Similarly, there was limited potential for improving animal model fit for full-sib family effects in the genetic analysis of survival data from BL2020 based on the result of likelihood ratio tests.

## Discussion

The current study provides the first genetic analysis of the trajectory of survival traits across grow-out stage for improved lines in a farmed aquatic species. Results of the genetic evaluation indicate that heritability estimates for the two defined types survival applied here were very similar. In general, estimated levels of heritability for survival traits were low but increased gradually across the grow-out test periods for both year classes. In addition, genetic correlations between the binary trait of survivability (S) and the continuous trait of survival time (SL) were relatively high. These findings will be useful for genetic analyses of survival data in field environments and potentially contribute to the development of relatively robust farmed aquatic strains used in aquaculture. An issue we identified in the analysis however, was missing records of some individual mortality events across our test periods. This reflects in general the difficulty of collecting survival data for aquaculture species in the field when working in commercial test environments.

### Mortality

Records of daily mortality in breeding lines are not only important for genetic evaluation of survival traits, but also for assessment of general husbandry management of population health status in breeding lines. Changes in daily mortality here were similar to those reported in our previous study with the mortality rate varying between 0.1% and 0.15% (Ren et al., 2020a). Designing and managing reliable, high quality water culture conditions is a major constraint in selective breeding programs for penaeid shrimps. Most daily mortality rates for farmed penaeids domestication programs range between 0.1% and 0.5%, depending on the different types of culture systems employed (Yano, 2000; Coman et al., 2005; Duy et al., 2012).

The highest daily mortality peaks in both test years occurred in late grow-out stages (Figure 1), periods that coincided with the coldest weather temperatures in the winter seasons at the hatchery location in China. In general, husbandry management of penaeid breeding lines are considered appropriate if daily mortality rate fluctuates around 0.5%, and higher mortality rates do not last for longer than 7–10 days. In our study, when mean daily temperatures increased from this relatively low range, mortality decreased to normal levels again. Furthermore, in most cases individuals that died during low temperature periods were of smaller or weaker individuals.

In the current study, we observed a gap in bias between survival estimators across the test period and the final population census data. Collecting survival data in natural ‘field’ conditions can be difficult in aquaculture breeding programs compared with that in terrestrial farm animals because most mortality events are under water and consequently, may not be detected. While survival phenotype derived from controlled experimental challenge tests can be more accurate (Ødegård et al., 2011; Yáñez et al., 2014), the use of survival data in natural field conditions can often provide better information for developing robust culture lines in aquaculture species (Houston et al., 2008; Lillehammer et al., 2013; Bangera et al., 2014; Dégremont et al., 2015). In particular, survival data collected under field conditions will better reflect natural processes of changes in mortality in a population and collecting these data can avoid potential G-by-E impacts when using artificial challenge test environments.

Survival estimators and final population census results in the current study were comparable with earlier reports on genetic breeding programs conducted on penaeid shrimps. In Mexico, survival rate of *P. vannamei* selection lines were reported to range from 71% to 82.2% during the grow-out test stages (Campos-Montes et al., 2013; Caballero-Zamora et al., 2015). Breeding nucleus data for farmed *P. vannamei* in Colombia reported survival rates ranging from 56.9% to 77.2% (Gitterle et al., 2005), while they were 70% and 73% for two grow-out stages in a selection program for *P. monodon* in Australia (Coman et al., 2010). In Vietnam, survival rates for *P. monodon* were 34%–49% in a family selection program (Van Sang et al., 2020).

### Animal models for survival analysis

While survival traits are often recorded as non-normally distributed binary traits, reports of genetic evaluation of survival data in dairy cattle (Sasaki et al., 2015; Van Pelt et al., 2015, 2016; Heise et al., 2016, 2018) suggest that linear models are likely to be more appropriate for genetic analysis of survival data in aquaculture. Proportion hazards models (PHM) have been often considered to fit time-to-event survival data better (Ducrocq, 1994; Neerhof et al., 2000), however, computing time to analyse these data successfully is much high than with linear models. Furthermore, using a PHM approach, it is difficult to assess genetic correlations with other continuous traits. Similarly, threshold models require significantly more computing resources for genetic analysis of survival data compared with linear models. Moreover, correlations of EBVs estimated with threshold models and linear models have been almost identical, indicating that there are few advantages of applying threshold models in routine genetic analyses of survival data (Boettcher et al., 1999; Vazquez et al., 2009). In practical terms, consideration of model choice in breeding programs can be a balance between multiple factors, including: simplified data records, comparative model performance, available computing resources, and integration of data from other important economic traits at the same time. We therefore consider linear multivariate animal models (LMA) to provide informative and reliable tools for routine genetic analysis of survival data in farmed aquatic species.

Data structure in the current study applied a larger number of full paternity and half-sib families that were suitable for achieving highly accurate heritability estimates. In addition, full and half-sib families were produced over a relatively short time period (1 week) that effectively removes time of age effects and common environmental effects in the genetic analysis. This can enhance the accuracy of the animal model results generated. We therefore chose a linear multivariate animal model (LMA) approach for our genetic analyses rather than other more sophisticated linear animal models such as a random regression model or repeatability model to fit age effects of time for the survival data set here.

### Heritability estimates

Heritability estimates for S1 to S12 and SL1 to SL 12 were consistent with results reported from other pacific white shrimp breeding programs. Gitterle et al. (2005) evaluated binary survival traits based on 430 full-sib families in different test environments and reported heritability estimates that ranged from 0.04 to 0.10. In another genetic analysis of 2008–2010 survival data of pacific white shrimp in Mexico, heritability estimates for early stage survival were 0.03 and 0.04 for later grow-out test stage (Campos-Montes et al., 2013). In a G-by-E effect study, heritability of survival was reported to be 0.06 under cold temperature conditions, while it was much higher (0.11) at normal ambient temperatures (Li et al., 2015). In addition, genetic parameters for survival in the presence or absence of a white spot disease outbreak were reported to be 0.00 and 0.06 (Caballero-Zamora et al., 2015), respectively. Overall, these results for heritability estimates of survival traits agree well with quantitative genetic theoretical predictions that survival traits are a group of fitness-associated traits that tend to show the lowest levels of heritability (Hill, 2010).

Here we compared two types of survival definitions; binary traits and continuous traits for our genetic evaluation, with both showing similar heritability estimates (Table 3). While this result did not fit our earlier assumption that continuous traits would improve the results for heritability estimates *via* adding more survival time information to mortality events, similar findings have been reported for genetic analysis of survival data in experimental challenge tests on aquaculture species (Joshi et al., 2021a; Vu et al., 2022). Binary trait recording however, is much more simple (0, 1) to score than continuous survival data in practice and thus, should be considered more feasible for routine genetic evaluation in aquaculture breeding programs.

Knowledge about heritability estimate trajectories across time is important to improve efficiency in selection programs and to provide insights on predicted selection response over the time period in the test. We believe that our study is the first report on the heritability of survival trajectory across grow-out stage in farmed aquatic species. Results here, suggest that for both lines and applying both types of survival trait definition, similar patterns for trajectory with time were evident. In general, initial heritability estimates were almost close to 0, following which they gradually increased across the grow-out test period before slowing down or reaching a plateau during the latter stages (Figure 2). Of interest, this pattern in trajectory is similar compared with some earlier reports for changes in growth heritability estimates across different age times, whereas heritability estimates for growth traits reached a plateau phase in the middle to later grow-out testing stage (Turra et al., 2012; He et al., 2017). In dairy science, similar patterns for survival trait heritability were also reported for a 72 month period of milk production life (Van Pelt et al., 2015). Therefore, this pattern of heritability estimate trajectories across time can be very useful for making critical decisions in a breeding plan.

Common full-sib effects (*c* ^*2*^) were not significant in the current study indicating that linear multivariate animal model (LMA) without full-sib effects was effective for routine genetic evaluation here. This finding agrees with a systematic review paper on *c* ^*2*^ estimation in aquaculture breeding suggests that full-sib effects contribute only a small proportion of total phenotypic variance at earlier growth stages for growth related traits, but for growth traits in an individual’s later stages or with other phenotypic traits, full-sib effects (*c* ^*2*^) are not significant in most cases and are essentially zero (Nguyen, 2021). As an example, routine genetic evaluation models applied in commercial tilapia selection breeding programs based on current published genetic animal models do not include full-sib effects (*c* ^*2*^) (Joshi et al., 2021b).

### Genetic and phenotype correlations

Both genetic and phenotypic correlations between binary and continuous survival traits time showed high correlations (>0.8) at each time window (Figure 2). In an aquaculture context, similar patterns have been reported for correlations between different types of growth related traits, including for *r*_*g*_ */r*_*p*_ among body weight, body length, and other morphological growth traits (He et al., 2017; Schlicht et al., 2018). Patterns for *r*_*g*_ */r*_*p*_ also support findings from heritability estimates that both survival definitions can be applied equally well for genetic selection on overall survival trait data in commercial breeding lines.

The current study is the first report on patterns of genetic correlations for survival traits across grow-out testing stage in a farmed aquatic species (Figure 3). In general, patterns of genetic correlation for survival traits across time (*r*_*g*_ among S1 to S12 and SL1 to SL12) in the current study were moderate to high. There was a trend here for a gradual decrease in *r*_*g*_ between survival traits with the time. These patterns for *r*_*g*_ were in accordance with trajectories of genetic correlations for growth traits across ages in other aquaculture studies (Turra et al., 2012; He et al., 2017; Schlicht et al., 2018), as well as for survival traits in livestock (Van Pelt et al., 2015). In addition, results for *r*_*p*_ trajectories of correlation across time between S1 to S12 and/or SL1 to SL12 were similar with that of *r*_*g*_, but values for *r*_*p*_ are always slightly lower than for *r*_*g*_ (Figure 3).

In contrast, genetic correlations between SUR and S1-12/SL1-12 were much lower but nevertheless, in most instances were still moderate (Figure 3). From observation of the patterns of survival traits across times, the real genetic correlations between SUR and other survival traits are likely to be much higher than that observed here. This most likely resulted from the missing data of survival records during the grow-out period and the final population census. Highly accurate recording of survival data in field commercial aquaculture environments however, is often difficult compared with data collection in challenge test experimental conditions, particularly, for pacific white shrimp that are of relatively small size and that are cannibalistic. These characteristics mean that some results of mortality events during grow-out test period are unlikely ever to be detected under field conditions.

In the current study, correlations between body weight and survival traits were generally low (Figure 3), but still positive, a result that is consistent with previous work on pacific white shrimp (Campos-Montes et al., 2013; Caballero-Zamora et al., 2015; Li et al., 2015; Ren et al., 2020a). This suggests overall, that survival and growth are two groups of separate traits and can be selected for simultaneously in a genetic breeding program *via* a multi-traits selection approach.

### Implications

While survival can be considered a relatively ‘old’ trait in animal breeding, often neglected because of the simple records (0 or 1) scored and generally low heritability estimates. Developing routine genetic evaluation systems for survival traits however, will be important for developing robust strains using valuable field survival data. Firstly, survival data collection from commercial lines can be used directly for improving overall survival rate in target breeding lines (Gjedrem and Rye, 2018). Moreover, survival data recorded from commercial lines when special events arise e.g., disease outbreaks can also be applied effectively to developing disease resistant strains, and these type of data are also valuable for avoiding potential G-by-E effects compared with survival data from controlled (artificial) challenge experiments (Bangera et al., 2014; Dégremont et al., 2015; Barría et al., 2020; Fraslin et al., 2022). Additionally, despite of low heritability of survival traits at the early grow-out testing stage, routine genetic evaluation of survival data in breeding lines provides a vital assessment of the relative health status of a stock under the general husbandry management practices employed in the breeding lines. Here we provide the first report of the trajectory of heritability estimates for survival traits across the grow-out period for pacific white shrimp breeding lines in China. The results will be useful for applying commercial field survival data to develop improved robust culture lines of this important crustacean species in the future.

## Conclusions

In conclusion, we developed routine genetic evaluations for survival data in selective breeding pacific white shrimp line in China. Results of heritability and genetic correlation estimates indicate that both survival definitions for binary traits and continuous traits of survival time can be used effectively for genetic analysis of survival data. Binary survival records following with linear multivariate animal models (LMA) provide a feasible routine genetic evaluation approach in practical commercial breeding programs because of the simple data recording, reduced computing time required, and the ability to combine performance assessment of multiple continuous phenotypic traits in the genetic analysis. While heritability estimates for survival traits here were generally low, they showed a gradual increasing trend across the grow-out period. Genetic correlations for survival traits during grow-out tests were moderate to high, and the closer the times were between estimates of two survival traits, the higher were the genetic correlations. Genetic correlations between survival traits and body weight were low but all estimates were positive. In summary, we first reported the trajectory of heritability estimates for survival traits across grow-out stage in aquaculture genetics, which would be useful for how to use survival data in field situations to develop more robust culture strains of *P. vannamei* and potentially for other farmed aquatic species in the future.

## Data Availability

The original contributions presented in the study are included in the article/Supplementary Material, further inquiries can be directed to the corresponding author.
